# Gelatin Methacryloyl (GelMA) Nanocomposite Hydrogels Embedding Bioactive Naringin Liposomes

**DOI:** 10.3390/polym12122944

**Published:** 2020-12-09

**Authors:** Kamil Elkhoury, Laura Sanchez-Gonzalez, Pedro Lavrador, Rui Almeida, Vítor Gaspar, Cyril Kahn, Franck Cleymand, Elmira Arab-Tehrany, João F. Mano

**Affiliations:** 1 LIBio, Université de Lorraine, F-54000 Nancy, France; kamil.elkhoury@univ-lorraine.fr (K.E.); laura.sanchez-gonzalez@univ-lorraine.fr (L.S.-G.); cyril.kahn@univ-lorraine.fr (C.K.); 2 Department of Chemistry, CICECO-Aveiro Institute of Materials, University of Aveiro, 3810-193 Aveiro, Portugal; pedrolavrador@ua.pt (P.L.); ruijorgealmeida@ua.pt (R.A.); vm.gaspar@ua.pt (V.G.); 3 Institut Jean Lamour, CNRS-Université de Lorraine, F-54000 Nancy, France; franck.cleymand@univ-lorraine.fr

**Keywords:** naringin, liposomes, human mesenchymal stem cells, GelMA, bone tissue engineering

## Abstract

The development of nanocomposite hydrogels that take advantage of hierarchic building blocks is gaining increased attention due to their added functionality and numerous biomedical applications. Gathering on the unique properties of these platforms, herein we report the synthesis of bioactive nanocomposite hydrogels comprising naringin-loaded salmon-derived lecithin nanosized liposomal building blocks and gelatin methacryloyl (GelMA) macro-sized hydrogels for their embedding. This platform takes advantage of liposomes’ significant drug loading capacity and their role in hydrogel network reinforcement, as well as of the injectability and light-mediated crosslinking of bioderived gelatin-based biomaterials. First, the physicochemical properties, as well as the encapsulation efficiency, release profile, and cytotoxicity of naringin-loaded nanoliposomes (LipoN) were characterized. Then, the effect of embedding LipoN in the GelMA matrix were characterized by studying the release behavior, swelling ratio, and hydrophilic character, as well as the rheological and mechanical properties of GelMA and GelMA-LipoN functionalized hydrogels. Finally, the dispersion of nanoliposomes encapsulating a model fluorescent probe in the GelMA matrix was visualized. The formulation of naringin-loaded liposomes via an optimized procedure yielded nanosized (114 nm) negatively charged particles with a high encapsulation efficiency (~99%). Naringin-loaded nanoliposomes administration to human adipose-derived stem cells confirmed their suitable cytocompatibility. Moreover, in addition to significantly extending the release of naringin from the hydrogel, the nanoliposomes inclusion in the GelMA matrix significantly increased its elastic and compressive moduli and decreased its swelling ratio, while showing an excellent dispersion in the hydrogel network. Overall, salmon-derived nanoliposomes enabled the inclusion and controlled release of pro-osteogenic bioactive molecules, as well as improved the hydrogel matrix properties, which suggests that these soft nanoparticles can play an important role in bioengineering bioactive nanocomposites for bone tissue engineering in the foreseeable future.

## 1. Introduction

The main goal of bone tissue engineering is to engineer cell-free or cell-rich biomaterial-based strategies that outperform the widely applied bone allografts and autografts [1]. In the context of cell-based therapies, human mesenchymal/stromal stem cells, either adipose (hASCs) or bone-marrow-derived (hBM-MSCs), arise as particularly attractive adult stem cell sources owing to their low immunogenicity, immunosuppressive and anti-inflammatory activities, ease of isolation via minimally invasive techniques, and multi-lineage differentiation potential (i.e., adipose, muscle, cartilage or bone tissue precursors) [2]. Considering hASCs differentiation toward osteogenic lineages, the widely explored pro-osteogenic differentiation strategies involving Dexamethasone (Dex) or bone morphogenetic protein type 2 (BMP–2) administration are often associated with reduced effectiveness and deleterious side effects, which limits their success as stimulatory bioactive molecules for cell-based therapies.

Recently, naturally available compounds that can potentially bioinstruct the pro-osteogenic lineage differentiation process are gaining particular interest due to their cytocompatibility and potent bioactivity. One of the most promising naturally available compounds is the citrus-derived phytotherapeutic naringin. This natural flavanone glycoside cannot only enhance the proliferation and differentiation of osteoprogenitor cells into osteoblasts but simultaneously inhibits osteoclastic activity [3]. Additionally, naringin presents well-established antioxidant, anti-inflammatory, anticancer, and antimicrobial activities that are beneficial in a wide array of biomedical strategies [3,4]. This flavanone offers several advantages when compared to recombinant BMP-2 or other synthetic pro-osteogenic pharmaceutics, such as repressing adipogenesis while solely promoting the osteogenic commitment of mesenchymal stem cells, and the ability to enhance the secretion of BMP-2 in osteoprogenitor cells while exerting a synergistic osteogenic effect with this osteoinductive protein [5,6,7,8,9].

Recent studies showcasing naringin’s multifunctional activities and specialized pro-osteogenic toolset underline the tremendous potential for this flavonoid in inducing stem cells osteodifferentiation [3,10,11,12,13]. However, like other natural compounds, naringin presents some drawbacks that limit its clinical efficacy, such as extensive metabolism upon administration and poor in vivo bioavailability [14,15]. To overcome such drawbacks, self-assembled lipid nanocarriers, that can encapsulate both hydrophobic and hydrophilic bioactive compounds, may be exploited to protect and deliver naringin, while aiming to maintain its therapeutic levels over extended periods [16]. Nanoliposomes produced from natural lecithin are natural lipid nanocarriers, composed of phospholipids that permit self-sealing in aqueous media [17,18,19,20,21]. They are biocompatible and have been vastly explored for applications in different fields including food [22], cosmetics [23], drug delivery [24,25,26], and tissue engineering [27,28,29]. Furthermore, the composition of the lipids used for nanoliposome bilayers is of special interest in augmenting cells and tissue response [30,31]. In this work, natural salmon lecithin used as the nanoliposome building block is highly enriched in mono and poly-unsaturated fatty acids (PUFAs), mainly linolenic acids (ω-3) and linoleic acids (ω-6), which have demonstrated to play an osteoprotective role by simultaneously preventing bone resorption and increasing bone mass in vivo [32,33]. Collectively, naringin-loaded soft nanoliposomes with intrinsic bioactivity have not been reported to date and appear highly promising candidates for bone tissue engineering strategies either as standalone systems or included in hydrogel networks.

The successful repair of critical bone defects depends on the interaction between the implant and the injured area. Hydrogels have been recently used for various tissue engineering applications due to their high water-binding capacity, porous structure, and tunable degradation properties [34]. Moreover, hydrogels’ shape can be tuned to different morphologies using different biofabrication techniques such as 3D bioprinting and fiber-based technologies [35]. From all different hydrogels previously used for bone tissue engineering applications, Gelatin methacryloyl (GelMA) hydrogel represents one of the most promising candidates for manufacturing scaffolds for bone tissue repair owing to its advantageous chemical tunability, biocompatibility, and promising in vitro and in vivo bone regeneration [36,37,38]. Also, in addition to gelatin being obtained from the denaturation of collagen, which is the major protein in bone tissues, recent results have shown that GelMA can significantly induce in vitro calcium deposition and osteogenic differentiation, as well as in vivo endochondral bone formation, having thus a great potential for being used in bone tissue engineering [39,40,41].

However, GelMA hydrogel networks are typically characterized by large internal pores, which allied to its hydrophilic nature, further hinders efficient loading of osteogenic hydrophobic compounds, and often leads to excessive burst release. Drugs are usually released within hours if they were encapsulated directly in GelMA hydrogels, whereas if they were loaded in liposomes before being embedded in the GelMA matrix, their release can be prolonged to several days [42]. In this context, embedding nanoparticles within the GelMA matrix overcomes the hydrogel’s poor drug loading and allows additional control over drug release kinetics, promoting a more sustained and prolonged release profile of the bioactive cargo [43]. Moreover, it has been reported that the inclusion of liposomes in the GelMA matrix enhances its strength, toughness, and flexibility, with no significant difference found between blank and loaded liposomes [44].

Gathering on this, herein we formulated an all-natural bioactive nanocomposite hydrogel comprising naringin-loaded salmon-derived nanoliposome building blocks and embedded in a photocrosslinkable GelMA hydrogel network. First, the optimization of manufacturing parameters and the physicochemical characterization of blank and naringin-loaded liposomes were performed. Afterward, the encapsulation efficiency, in vitro release, and biocompatibility of encapsulated naringin were evaluated. In addition to the loaded-liposomes’ dispersion in the GelMA matrix, their effect on the swelling behavior and the surface, rheological, mechanical properties of the GelMA matrix was investigated.

## 2. Materials and Methods

Human adipose-derived mesenchymal/stromal stem cells (hASCs, ATCC PCS-500-011^®^) were purchased from LGC Standards S.L.U. (Barcelona, Spain). Naringin (>95% purity) and Amicon Ultra-4 mL (100 kDa molecular weight cut-off (MWCO) filters were all purchased from Laborspirit (Lisbon, Portugal)). Salmon lecithin was obtained by enzymatic hydrolysis as described by Linder et al. [45]. A low-temperature enzymatic methodology was used to extract lipidic fractions, without requiring any organic solvents. Float-A-Lyzer G2 (3500–5000 Da MWCO) dialysis membranes were purchased from Reagente 5 (porto, Portugal). Fetal bovine serum (FBS, E.U. approved, South American origin), minimum Essential Medium α-modification (α-MEM), antibiotic, and alamarBlue^®^ were purchased from Alfagene (Lisbon, Portugal). 3,3′-Dioctadecyloxacarbocyanine perchlorate (DiO), and Dulbecco’s PBS (dPBS) were purchased from Thermo Fisher Scientific (Oeiras, Portugal). Gelatin from porcine skin (Type A, 300 bloom), methacrylic anhydride (MA), Irgacure 2959 (PI) (2-hydroxy-4′-(2-hydroxyethoxy)-2-methylpropiophenone), and 2,4,6-trinitrobenzenesulfonic acid solution (TNBS) were all purchased from Sigma Aldrich (Saint-Quentin-Fallavier, France).

### 2.1. Preparation of Blank and Loaded Nanoliposomes

For nanoliposomes formulation, 200 mg of salmon lecithin were dissolved in 9.8 mL of distilled water. The suspension was then mixed for 4 h under stirring in an inert atmosphere (nitrogen) and then sonicated at 40 kHz and 40% of full power for 240 s (1 s on and 1 s off cycles) to obtain a homogeneous solution of blank nanoliposomes.

For producing drug-loaded liposomes the film hydration method was employed. In brief, 200 mg of salmon lecithin and 100 mg of naringin were dissolved in a mixture of chloroform-methanol at a ratio of 2:1 (9 mL). Afterward, the lipid-drug solution was included in a round-bottom flask and transferred to a rotary evaporator (Rotavapor^®^ Büchi R-300, Büchi Labortechnik AG, Switzerland). After complete evaporation of organic solvents under vacuum, a thin lipid film was formed. Thin-film hydration was promoted (9.8 mL of distilled water) and the resulting suspension was stirred at 800 rpm for 4 h under a nitrogen atmosphere. The samples were then sonicated at 40 kHz and 40% of full power for 240 s (1 s on and 1 s off) to obtain a homogeneous colloidal dispersion of naringin-loaded nanoliposomes.

### 2.2. Nanoliposomes Characterization

The average hydrodynamic particle diameter (Hd), polydispersity index (PDI), and ζ-potential of the prepared blank and drug-loaded nanoliposomes were characterized by DLS with a Zetasizer Nano ZS equipment (Malvern Instruments Ltd., Malvern, UK). Prior to measuring size and ζ-potential, the samples were diluted (1:200) with ultrapure distilled water. Measurements were performed at 25 °C with a fixed scattering angle of 173°, the refractive index (RI) at 1471, and absorbance at 0.01. The measurements were performed in standard capillary electrophoresis cells equipped with gold electrodes (DTS 1070). At least three independent measurements were performed for each condition. Blank and loaded nanoliposomes colloidal stability was examined by observing changes in size, PDI, and ζ-potential upon storage. Nanoliposomes were characterized via DLS at different time points, namely 0, 20, and 40 days.

### 2.3. Transmission Electron Microscopy (TEM) Analysis

Blank and naringin-loaded nanoliposomes structures were observed using transmission electron microscopy (TEM) via a negative staining method according to Colas et al. protocol [46]. Briefly, to reduce the concentration of nanoliposomes, samples were diluted with ultrapure distilled water (25-fold). To stain nanoliposomes, equal volumes of the diluted solution and an aqueous solution of ammonium molybdate (2%), used as a negative staining agent, were mixed. After the staining procedure, samples were kept at room temperature for 3 min, followed by a 5 min incubation on a copper mesh coated with carbon. Finally, samples were observed using a CM20 TEM (Philips, Amsterdam, Netherlands) associated with a TEM CCD camera (Olympus, Tokyo, Japan).

### 2.4. Encapsulation Efficiency

The encapsulation efficiency (EE) of naringin was obtained via ultraviolet-visible (UV-vis) absorbance of its peak (λ = 282 nm) which corresponds to the benzoyl moiety. Briefly, after naringin-loaded nanoliposomes were prepared, they were dialyzed with Float-A-Lyzer G2 dialysis devices (3.5–5 kDa MWCO) in 40 mL dPBS for 3 h and analyzed by UV-vis at λ = 282 nm. Dialyzed blank nanoliposomes established the control for UV-vis quantification. The absorbance was measured in a quartz microplate (Hellma^TM^ transparent 96-well quartz plate, VWR, Lisbon, Portugal) using a microplate reader equipped with a tungsten halogen lamp (Synergy HTX Biotek, Izasa Scientific, Carnaxide, Portugal). A calibration curve of naringin in dPBS was created to quantify the EE. EE was calculated using Equation (1):

EE (%) = (1 − Nr/Ni) × 100(1)

where EE (%) is the encapsulation efficiency, Nr the amount of naringin present in the dialysate and Ni the initial amount of naringin added.

### 2.5. In Vitro Release Profile of Naringin Loaded in Nanoliposomes

To mimic the physiological scenario, the in vitro release profile of naringin was examined in dPBS at pH 7.4. Briefly, 2 mL of freshly produced naringin-loaded nanoliposomes was transferred to the Float-A-Lyzer G2 dialysis device and submerged in 40 mL of dPBS. The release profile was investigated at 37 °C and stirred at 600 rpm. At specific time points (1 h, 2 h, 3 h, 24 h, 48 h, 72 h, 96 h, 120 h, 144 h, 168 h, and 192 h), 1 mL of samples was recovered from the dialysate and replaced by 1 mL of fresh dPBS. A standard naringin calibration curve in dPBS was used to quantify the cumulative release.

### 2.6. Cytotoxicity and Cellular Proliferation Assays

Cells were maintained in a humidified 5% CO_2_ incubator at 37 °C and manipulated in a biosafety cabinet. hASCs were routinely cultured in basal culture medium (BM) comprised of α-MEM supplemented with 1% *v*/*v* of an antibiotic mixture and 10% *v*/*v* heat-inactivated FBS. BM was exchanged every 3–4 days. Cells were subcultured before reaching confluence.

AlamarBlue^®^ cell viability assay was used to evaluate the cell metabolism of hASCs. Cells were seeded in a 96-well plate overnight in BM at a density of 3.5 × 10^3^ cells per well (*n* = 10). Later, cells were incubated with BM containing naringin-loaded liposomes at concentrations of 25 μg mL^−1^, 50 μg mL^−1^, and 100 μg mL^−1^ of naringin. The medium was switched to BM and alamarBlue after 24 h and 96 h. The media was then transferred to a black, clear-bottom 96-well plate and resorufin fluorescence was quantified at an excitation/emission of λ_ex_ = 540 nm/λ_em_ = 600 nm, in a multimode microplate reader.

### 2.7. GelMA Synthesis and Hydrogels Preparation

GelMA was synthesized, from porcine skin gelatin type A, according to the general method first adopted by Van Den Bulcke et al. [47]. In brief, gelatin was mixed at 10% (*w*/*v*) into dPBS at 60 °C and stirred until fully dissolved. Then, 0.6 g of MA/1 g of gelatin was added dropwise to the gelatin solution, at 50 °C and a rate of 0.5 mL min^−1^ under stirring and allowed to react for 1 h. After 1 h, the reaction was stopped following 5× dilution with warm (40 °C) dPBS. To remove salts and unreacted MA, the mixture was dialyzed for 5 days at 40 °C against distilled water using 12–14 kDa cutoff dialysis tubing, in the dark. The solution was finally freeze-dried, generating a porous white foam that was stored at −20 °C until further use. The degree of substitution (D.S.) was determined by the TNBS assay [48], (D.S.: 76.4 ± 1.1%, *n* = 3).

GelMA solution was prepared by dissolving 10% (*w*/*v*) of the freeze-dried biopolymer into a dPBS solution at 50 °C. Then, 0.5% (*w*/*v*) of PI was added and the temperature was increased to 70 °C to reach complete solubilization. GelMA solution was UV crosslinked (360–480 nm) in a specific PDMS mold for 40 s to create hydrogel discs. Nanocomposite GelMA hydrogels were prepared using the same protocol by mixing naringin-loaded liposomes at a concentration of 50 μg mL^−1^ of naringin with the GelMA solution before UV crosslinking.

### 2.8. In Vitro Release Profile of Naringin Embedded in GelMA

To study the release profile, naringin and naringin-loaded nanoliposomes where embedded in GelMA discs that were transferred to 12–14 kDa cutoff dialysis tubing containing 5 mL of dPBS and submerged in 15 mL of dPBS. The release profile was investigated at 37 °C and stirred at 600 rpm. At specific time points (1 h, 2 h, 3 h, 24 h, 48 h, and 72 h), 2 mL of samples was recovered from the dialysate and replaced by 2 mL of fresh dPBS. A standard naringin calibration curve in dPBS was used to quantify the cumulative release.

### 2.9. Mass Swelling Ratio

The mass swelling ratio was evaluated by using five cylindrical samples (2 cm diameter, 2 mm height). The samples were kept in dPBS at 37 °C for 24 h. The excessive water was gently removed using a paper tissue, and the swollen weight of the samples was measured using a precision balance. Later, the samples were freeze-dried for 3 days and their dry weight was measured. The mass swelling ratio was calculated as the ratio of the mass value after swelling to the mass value of dried samples after lyophilization.

### 2.10. Surface Properties

The polar component (γ^P^), dispersive component (γ^D^), and the surface tension (γ) of hydrogels were quantified according to the Owens and Wendt method [49]. This quantification was achieved using water (γ_Liq_ = 72.8 mN m^−1^; γ_Liq_^D^ = 21.8 mN m^−1^; γ_Liq_^P^ = 51 mN m^−1^), diiodomethane (γ_Liq_ = 50.8 mN m^−1^; γ_Liq_^D^ = 50.8 mN m^−1^; γ_Liq_^P^ = 0 mN m^−1^), and glycerol (γ_Liq_ = 63.4 mN m^−1^; γ_Liq_^D^ = 37 mN m^−1^; γ_Liq_^P^ = 26.4 mN m^−1^) according to the following Equations (2) and (3):

(2) $$\gamma =\gamma^{D}+\gamma^{P}$$

(3) $$\gamma_{Liq}\left(1+\cos\theta \right)=2\left(\sqrt{\gamma^{D}\;\times \gamma_{\mathrm{Liq}}^{D}}+\;\sqrt{\gamma^{P}\;\times \gamma_{\mathrm{Liq}}^{P}}\right)$$

where θ, γ, γ^P^, γ^D^, γ_Liq_, γ_Liq_^D^, and γ_Liq_^P^ are the contact angle, the surface tension, the dispersive and the polar components of the hydrogel’s surface and the tested liquid. All the surface tension parameters are expressed in mN m^−1^ and the contact angle is expressed in degree.

The contact angle measurements were carried out using three liquids (water, diiodomethane, and glycerol), with well-known polar and dispersive components via the sessile drop method on a goniometer (Drop Shape Analyzer 30, KRÜSS GmbH, Hamburg, Germany). First, a 2 μL droplet of each liquid was deposited on the hydrogel surface with a precision syringe. Then, the contact angle was measured between the tangent at the drop boundary and the baseline of the water drop. Three measurements per hydrogel were carried out.

### 2.11. Rheological Testing

Amplitude sweep tests at a frequency of 1 Hz were performed using a Kinexus pro rheometer (Malvern Instruments Ltd., Worcestershire, UK) equipped with a plane-plane geometry with a diameter of 20 mm. The hydrogel was loaded into a 1 mm gap between the plates and allowed to relax until the normal force was zero. The amplitudes of shear stress were carried out over a pressure range from 0.01 Pa to 500 Pa. The test was done with a constant frequency of 1 Hz at 37 °C and the measuring system was covered with a humid chamber to minimize the evaporation of the water. Three different hydrogel discs are tested for each type of hydrogel with the same experimental parameters.

### 2.12. Mechanical Testing

The mechanical measurements were performed using a universal testing machine (Lloyd-LRX, Lloyd Instruments, Fareham, UK). The samples were prepared in a cylindrical shape (2 cm diameter and 2 mm height). Compression tests were performed at a crosshead speed of 1 mm/min until fracture occurred. Prior to all measurements, the zero-gap was determined. Five samples of each condition were tested. The compressive modulus was determined as the slope of the linear region corresponding to the elastic part (10–20% strain) of the stress-strain curve.

### 2.13. 3D Bioprinting and Confocal Laser Scanning Microscopy Analysis

GelMA embedded, DiO-loaded nanoliposomes bioink (containing Irgacure 2959 photoinitiator, 0.1% in PBS pH = 7.4) was used to 3D bioprint disc-shaped constructs using a pneumatic extrusion bioprinter INKREDIBLE+ (CELLINK, Gothenburg, Sweden) equipped with a 23G nozzle, operating at pressures ranging from 60–70 kPa. GelMA-nanoliposomes were UV crosslinked (360–480 nm) for 40 s to generate nanocomposite hydrogels (Omnicure S-2000, 0.86 W/cm^2^). Confocal laser scanning microscopy imaging was performed in an LSM 880 Airyscan microscope (Carl Zeiss, Oberkochen, Germany) equipped with GaAsP/PMT detectors and a 20x/NA 0.8 Plan-Apochromat objective. Acquired data was post-processed in Zeiss ZEN v2.3 blue edition software.

## 3. Results and Discussion

### 3.1. Nanoliposomes Formulation and Characterization

**Figure 1:**
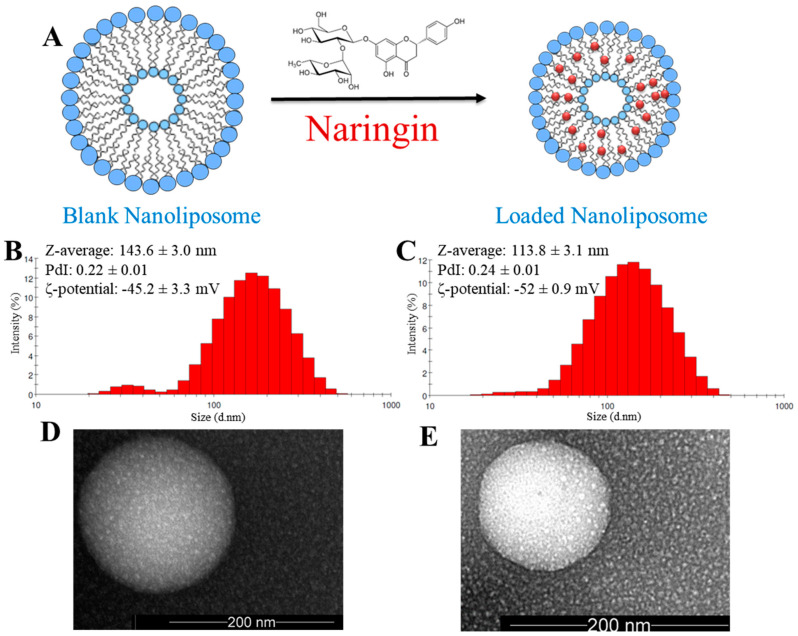
(**A**) Schematic representation of blank and naringin-loaded nanoliposomes. Physicochemical characterization via DLS of (**B**) blank and (**C**) loaded nanoliposomes. TEM images of (**D**) blank and (**E**) loaded nanoliposomes.

The applied sonication parameters during the naringin loading process (Figure 1A) can affect the minimum size that can be achieved. The size of the naringin-loaded nanoliposomes (~114 nm) was found to be smaller than that of the blank nanoliposomes (~144 nm) (Figure 1B,C), which suggests the presence of a strong interaction between naringin and salmon lecithin nanoliposomes that leads to core compaction [20,50]. The size was further confirmed with the TEM images (Figure 1D,E) that also revealed liposomes spherical morphology. The formulated liposomes were relatively monodisperse, presenting a low PdI (<0.25) associated with a narrow size distribution [51]. For nanoliposomal formulations used for drug delivery applications, a PDI value lower than 0.3 indicates a homogenous population and is considered to be acceptable [52]. ζ-potential for both blank (−45 mV) and loaded (−52 mV) nanoliposomes was negative, which is probably caused by the negatively charged phospholipids of salmon lecithin, such as phosphatidylserine, phosphatidic acid, phosphatidylglycerol, and phosphatidylinositol that can be exposed at the nanoliposomes surface [28].

Particle size, polydispersity index (PdI), and ζ-potential of blank and naringin-loaded nanoliposomes were measured directly after preparation, as well as after 20 and 40 days to assess their colloidal stability.

The results presented in Table 1 indicate that throughout the storage period nanoparticles’ size increased but stayed in the nanometric range (<200 nm), the ζ-potential decreased for higher negative values, and the particles maintained their relative narrow distribution with PdI values <0.3. Nanoliposomes stability was probably due to the high negative ζ-potential value because the higher this value, the stronger the repulsion between two adjacent particles is, and thus the higher the stability of the nanoliposomal formulation will be.

### 3.2. Encapsulation Efficiency and In Vitro Release Profile of Naringin Loaded in Nanoliposomes

Nanoliposomes achieved a high EE of naringin (99.7 ± 0.07%) that was quantified by UV-vis analysis and extrapolated from naringin’s calibration curve. This encapsulation efficiency was consistent with other studies using liposomes to encapsulate naringin [50] and its aglycone derivative, Naringenin [53]. Nanoliposomes achieved a higher EE of naringin than that of methoxy-poly(ethylene glycol)-maleimide-thiol-poly(l-lactide) (mPEG-MS-PLA) polymeric nanomicelles which achieved an EE of 87.2 ± 4.6% [10].

**Figure 2:**
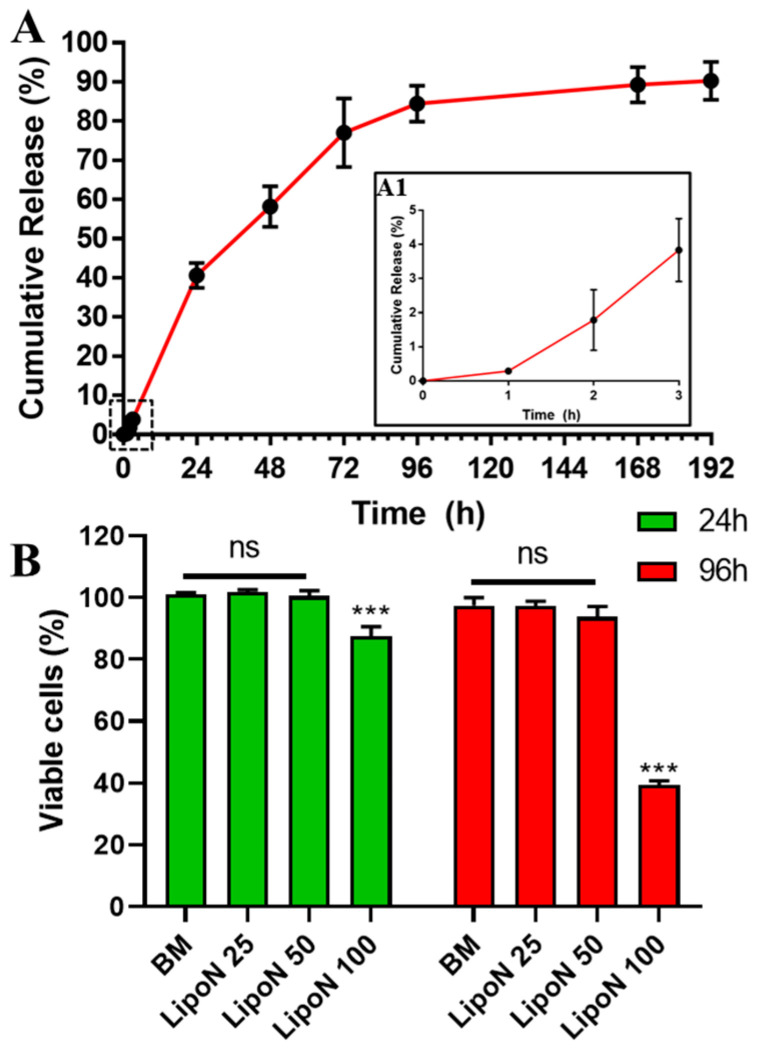
(**A**) Naringin-loaded nanoliposomes in vitro cumulative release profile in dPBS (pH = 7.4) at 37 °C. (**A1**) The zoomed section representing the cumulative release during the first 3 h. Data are presented as mean ± s.d. (*n* = 3). (**B**) hASCs cell viability at 24 and 96 h following incubation with basal culture medium (BM) and different naringin concentrations (25, 50, and 100 μg mL^−1^) loaded in nanoliposomes (LipoN, 50,). Data are represented as mean ± s.d., *n* = 5. The reported data were analyzed using a two-way ANOVA followed by Holm-Sidak’s test. Significance was indicated as *** (*p* < 0.001).

A biphasic release was observed for naringin-loaded nanoliposomes (Figure 2A). The release profile showed a slow release of about 4% only within the first three hours, followed by sustained drug release over the next 8 days. The obtained release profile is comparable to those obtained in the literature [10]. Interestingly, the salmon derived nanoliposomes exhibited only a 40% release after 24 h, suggesting that nanoliposomes provide a more sustained release compared to previously reported polymeric nanomicelles (~65% at 24 h) [45]. This suggests that salmon-derived lipids prevent the initial burst release and provide a controlled extended release of the encapsulated flavonoid. This can improve naringin’s in vivo bioavailability and stability, which might lead to improvements in its therapeutic effect, and prevent any unwanted interactions with other molecules [3,50].

Previously, a significant pro-osteogenic effect between free and encapsulated naringin was observed [3,10]. These findings suggest that the controlled release of this flavonoid might affect the promotion of osteodifferentiation. This osteodifferentiation can be further increased by the high percentage of ω-3 PUFAs present in salmon nanoliposomes. Previously, these nanoparticles have been found to be composed of around 50% PUFAs, of which about 10% EPA and 24% DHA, and an ω-3/ω-6 ratio of 3.8 [18]. These ω-3 PUFAs have been found to be able to stimulate the expression of Cbfa1 transcription factor involved in the initiation and modulation of osteoblast differentiation [54,55]. Moreover, ω-3 PUFAs can regulate bone metabolism, decrease osteoclastogenesis, and modulate the number of proinflammatory cytokines which improves calcium accretion in bone [56]. The high ratio of ω-3 to ω-6 can protect against the bone mass loss, since it was found that ω-6 diminishes the *opg/rankl* gene expression in osteoblasts which stimulates MSC differentiation into adipocytes, favors the osteoclastic activity, and reduces the production of osteoblasts [57]. So, these ω-3 rich nanoliposomes can protect the bones formed by the osteodifferentiation of hASCs induced using naringin

### 3.3. Cellular Viability

Although salmon nanoliposomes have been found to be cytocompatible toward cortical neurons and Wharton’s jelly human stem cells [20,21,28], investigating a possible cytotoxic response with a new cell type is an important requirement. Naringin has already been tested for its cytotoxicity toward hASCs in the range of 5–50 μg mL^−1^ [10]. However, evaluating the cytotoxicity of naringin-loaded in nanoliposomes is important for the envisioned final application as bone regenerative nanocomposite hydrogels. For this hASCs were incubated with different concentrations of naringin-loaded nanoliposomes (LipoN 25, 50, and 100), corresponding to naringin concentrations of 25, 50, and 100 μg mL^−1^ and nanoliposomes concentrations of 50, 100, and 200 μg mL^−1^. Results show that LipoN 25 and LipoN 50, contrary to LipoN 100, showed no cytotoxicity at 24 h and 96 h (Figure 2B). These findings are in complete agreement with previous studies. Naringin has been found to be biocompatible and to significantly induce cell proliferation of osteoprogenitor cells [8,58,59]. Whereas, Mahmoud et al., Latifi et al., and Dostert et al., previously reported the biocompatibility of low concentrations of salmon-derived nanoliposomes [20,21,28]. These results suggest that naringin-loaded salmon-derived nanoliposomes with a maximum naringin concentration of 50 μg mL^−1^ (LipoN 50) present high biocompatibility toward hASCs. Having confirmed this parameter, the nanosized blocks, LipoN 50, were embedded in GelMA and subjected to UV-mediated photocrosslinking to yield naringin-loaded nanocomposite hydrogels (GelMA-LipoN).

### 3.4. In Vitro Release Profile of Naringin Embedded in GelMA

**Figure 3:**
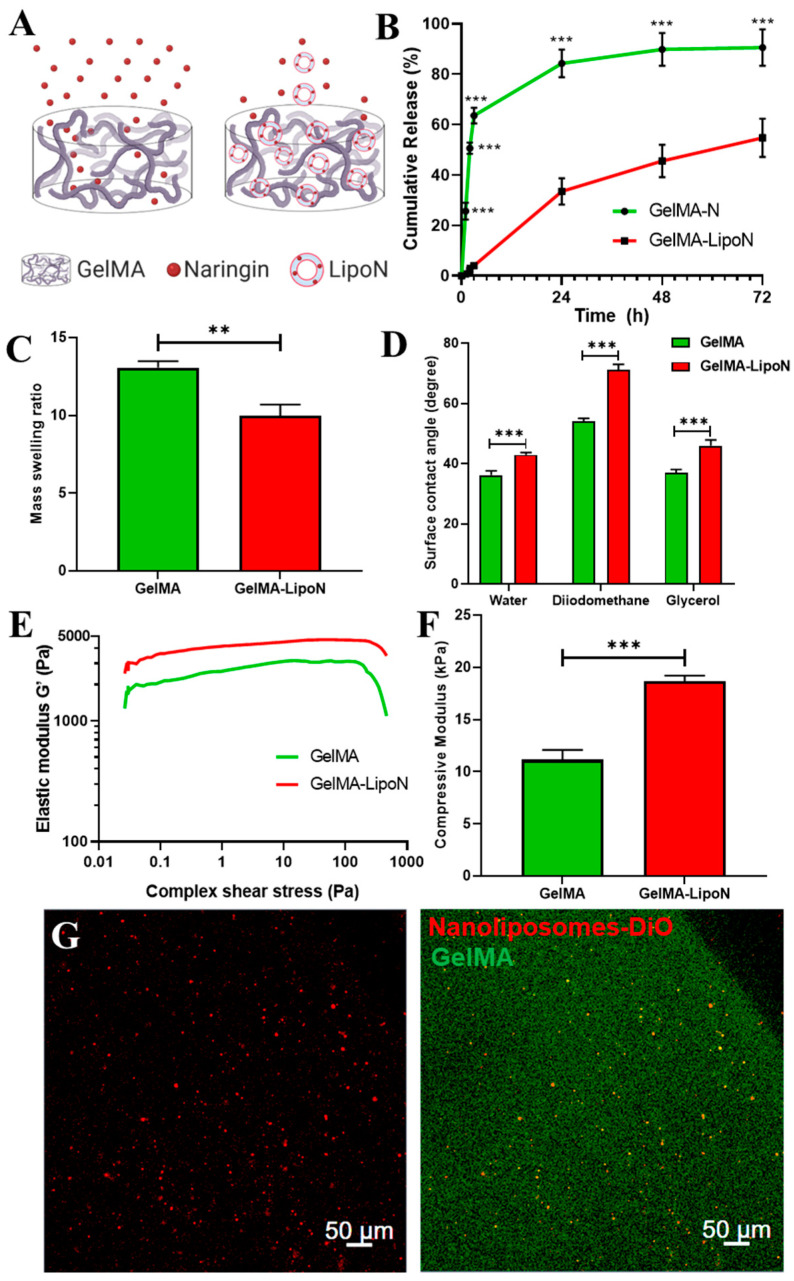
(**A**) Schematic representation of the different release behavior of naringin embedded directly in GelMA (GelMA-N) versus when first encapsulated in liposomes (GelMA-LipoN). Created with BioRender.com. (**B**) The in vitro cumulative release profile of GelMA-N and GelMA-LipoN in dPBS (pH = 7.4) at 37 °C. (**C**) The mass swelling ratios of GelMA and GelMA GelMA-LipoN hydrogels. (**D**) The surface contact angle of GelMA and GelMA-LipoN hydrogels measured using water, diiodomethane, and glycerol. (**E**) The elastic modulus (G’) as a function of complex shear stress and (**F**) the compressive modulus of GelMA and GelMA-LipoN hydrogels. (**G**) Confocal micrographs of DiO-loaded nanoliposomes embedded in a bioprinted 3D GelMA construct. Red channel: nanoliposomes-DiO. Green Channel: GelMA autofluorescence. Data are represented as mean ± s.d., *n* = 3. The reported data of (**C**,**F**) were analyzed using a Student’s *t*-test and of (**B**,**D**) using a two-way ANOVA followed by Holm-Sidak’s test. Significance was indicated as ** (*p* < 0.01) and *** (*p* < 0.001).

GelMA-N (GelMA hydrogels embedding free naringin) and GelMA-LipoN presented different release behaviors and profiles (Figure 3A,B), as the nanocomposite GelMA hydrogels released significantly fewer quantities of naringin than the liposome-free GelMA hydrogels. This might be due to the small size of the phytotherapeutic that can easily pass through the microsized pores of GelMA. According to the results, the amount of naringin released from GelMA-LipoN was significantly extended compared to GelMA-N. GelMA-N released 65% of naringin in the first 3 h, compared to the ~4% released by GelMA-LipoN. At 72 h, less than 10% of naringin remained in GelMA-N compared to more than 45% in nanocomposite hydrogels. The obtained release profiles of GelMA and nanocomposite GelMA are comparable to those obtained in the literature for naringin and other therapeutic molecules [42,60,61]. This suggests that GelMA scaffolds can be successfully loaded with LipoN and provide a controlled extended release of the encapsulated flavonoid, which might improve its in vivo bioavailability and hence its therapeutic effect.

### 3.5. Swelling Behavior

The swelling behavior of crosslinked nanocomposite hydrogels is an essential parameter, as it affects the diffusion of solutes and the mechanical properties of these platforms along time [62]. The swelling behavior is governed by the crosslinking density, hydrophilicity, and structural properties of the hydrogel, as well as by its interactions with the solvent [63,64]. To study this behavior, GelMA and GelMA-LipoN were fully swelled in dPBS at 37 °C for 24 h to obtain their swollen weight and then freeze-dried for three days to obtain their dry weight. Using the swollen and dry weight of GelMA, the mass swelling ratio was then calculated. GelMA had a higher mass swelling ratio than GelMA-LipoN (Figure 3C). The decrease in swelling ratio of GelMA-LipoN hydrogels is considered an advantageous property since scaffolds will undergo a limited shape transformation once contacted with body fluids [42]. The low swelling ratio may also assure a higher residence time of the nanoliposomes in the nanocomposite hydrogels matrix by preventing their premature clearing. A plausible explanation for this decrease in the mass swelling ratio is that the level of intermolecular crosslinking through noncovalent forces (electrostatic interactions or hydrogen bonds) have increased when nanoliposomes were embedded in GelMA. These hydrogen bonds can be created between phosphorous or other liposomal molecules and nitrogen or other elements in the GelMA network. Those newly formed bonds will increase the micro-crosslinking level of GelMA, which will thus lead to a decrease in pores’ size and in the volume of retained fluid, since hydrogels with smaller pores are able to withhold a smaller volume of fluid [62,63,65].

### 3.6. Contact Angle

The water contact angle on the GelMA hydrogel was significantly lower than on the GelMA-LipoN hydrogel (36.2° ± 1.4° vs. 42.9° ± 0.7°, Figure 3D), revealing a much lower water affinity of the GelMA-LipoN’s surface. Such behavior can be attributed to the newly formed hydrogen bonds on the GelMA surface following loaded-nanoliposomes embedding which will not only have a repulsive reaction to the droplet of water but also reduce the porosity which will lead to lower capillarity forces, and thus decrease water absorption. Indeed, the surface free energy of the GelMA was increased after the introduction of the loaded-liposomal soft nanoparticles (Table 2), due to a higher polar contribution. All in all, the embedment of loaded-nanoliposomes decreased the hydrophilic character of the GelMA matrix, which means that the GelMA-LipoN scaffolds offer better barrier properties.

### 3.7. Rheological and Mechanical Properties

Rheological measurements were carried out to assess the elastic properties of GelMA hydrogels. Results, presented in Figure 3E, show that the elastic modulus G’, also known as real modulus or storage modulus, of GelMA hydrogels (~2000 Pa) was lower than G’ of GelMA-LipoN hydrogels (~3500 Pa). Moreover, to investigate the impact of the integration of nanoliposomes in the GelMA matrix on the hydrogel’s mechanical properties, a compression test was performed on hydrogel samples. Figure 3F shows the compressive modulus that was calculated from the slope of the linear region corresponding to the elastic part (10–20% strain) of the stress-strain curve. It can be seen that the integration of nanoliposomes has led to a significant increase in the compression modulus from 11.14 ± 0.94 kPa for GelMA to 18.87 ± 0.53 kPa for GelMA-LipoN. The higher elastic and compression moduli of GelMA-LipoN might be caused by the newly formed hydrogen bonds that can produce a double crosslinked structure and thus can sustain higher external stress and load. Gen et al., previously reported a higher elastic modulus in GelMA hydrogels embedding liposomes [44]. Wu et al., previously reported a similar significant increase in the compressive modulus following liposomes integration in the GelMA matrix [42]. Creating composite hydrogels with improved mechanical properties is of great importance for bone tissue engineering applications since these scaffolds will provide a temporary structural and mechanical support to the laden proliferating and differentiating stem cells, leading to the synthesis of mineralized bone matrices that will replace the scaffold itself [66].

### 3.8. Nanoliposomes Dispersion in Nanocomposite Hydrogels Matrix

The confocal micrographs of the GelMA-LipoN (Figure 3G) show that nanoliposomes encapsulating DiO presented a good dispersion within 3D bioprinted GelMA scaffolds without any significant aggregation being visualized. In addition to the controlled release of naringin, the nanoliposomes stability upon dispersion in complex protein-based mixtures is another valuable property, that can assure the equal presence of the drug encapsulated inside the nanocomposite platforms to all encapsulated stem cells, which can maximize its effect. Similar liposomal distribution was previously observed in chitosan/gelatin hydrogels by Ciobanu et al. [67]. This homogeneous distribution can be very valuable for nanocomposite platforms engineering with the purpose of being applied in tissue engineering when used as implantable controlled delivery systems or as cell-laden scaffolds.

## 4. Conclusions

Throughout this study, the development and characterization of a potential bone regenerative nanocomposite hydrogel were achieved. The nanofunctionalized platform was comprised of a naturally available drug encapsulated in salmon-derived nanoliposomes and embedded in modified gelatin hydrogels. The inclusion of naringin in nanoliposomes resulted in a high encapsulation efficiency and a controlled drug release profile. The loaded nanoliposomes (LipoN 25 and LipoN 50) showed no cytotoxicity toward hASCs. Taken together with previous studies, the findings of this study provide evidence that nanoliposomes loaded with naringin are highly biocompatible and can be safely used for bone tissue engineering applications. Since nanoliposomes are derived from salmon fish, they are rich in ω-3 PUFAs which offers a double functionality of being bioactive on their own and being able to encapsulate bioactive molecules, increasing further their efficacity.

Moreover, the embedment of nanoliposomes in GelMA has not just improved its mechanical and rheological properties but also decreased its swelling ratio and hydrophilic character and extended the release of naringin, so nanoliposomes have toughened the GelMA scaffold, made it more resistant to shape transformations, and improved its barrier properties and drug release behavior. To add to all these advantages, nanoliposomes did not form any aggregates and had a homogeneous distribution in the GelMA construct after the bioprinting process, which suggests that the natural nanocomposite hydrogel composed of naringin-loaded nanoliposomes embedded in GelMA may be a promising bioink candidate for the bioprinting of cell-laden bioactive pro-osteogenic constructs. Since GelMA printed structures suffer from a poor resolution [68], the developed nanocomposite hydrogel will be further evaluated and optimized as a bioink to balance between its printability and functionality. Future studies will focus also on the ability of naringin-loaded nanoliposomes to induce the osteodifferentiation of hASCs in 2D cell culture, as well as in 3D cell culture when encapsulated inside the GelMA hydrogel matrix.

## Figures and Tables

**Table 1:** Blank nanoliposomes (BL) and naringin-loaded nanoliposomes (LL) mean particle size, polydispersity index (PdI), and ζ-potential at day 0, 20, and 40.

	Day	BL	LL
Size (nm)	0	143.6 ± 3.0	113.8 ± 3.1
	20	148.6 ± 5.2	131.0 ± 1.3
	40	168 ± 9.9	152.1 ± 1.6
PdI	0	0.22 ± 0.01	0.24 ± 0.01
	20	0.27 ± 0.01	0.29 ± 0.01
	40	0.28 ± 0.02	0.27 ± 0.02
ζ-potential (mV)	0	−45.2 ± 3.3	−52.0 ± 0.9
	20	−49.2 ± 0.9	−53.8 ± 0.4
	40	−56.9 ± 1.0	−54.8 ± 0.4

**Table 2:** The surface tension of hydrogels (γ) and its polar (γP) and dispersive (γD) components.

	GelMA	GelMA-LipoN
γ^P^ (mN m^−1^)	28.8 ± 0.6	31.7 ± 1.1
γ^D^ (mN m^−1^)	29.2 ± 0.5	21.2 ± 1.7
γ (mN m^−1^)	58.0 ± 0.4	52.9 ± 2.0
